# Cardiac Patch Transplantation Instruments for Robotic Minimally Invasive Cardiac Surgery: Initial Proof-of-concept Designs and Surgery in a Porcine Cadaver

**DOI:** 10.3389/frobt.2021.714356

**Published:** 2022-01-18

**Authors:** Christopher D. Roche, Gautam R. Iyer, Minh H. Nguyen, Sohaima Mabroora, Anthony Dome, Kareem Sakr, Rohan Pawar, Vincent Lee, Christopher C. Wilson, Carmine Gentile

**Affiliations:** ^1^ Northern Clinical School of Medicine, Kolling Institute, University of Sydney, Sydney, NSW, Australia; ^2^ Faculty of Engineering and IT, University of Technology Sydney (UTS), Sydney, NSW, Australia; ^3^ Department of Cardiothoracic Surgery, University Hospital of Wales, Cardiff, United Kingdom

**Keywords:** robotics, keyhole surgery, minimally invasive (MIS), cardiac surgery, myocardial repair, cardiac patch, thoracic, cardiothoracic

## Abstract

**Background:** Damaged cardiac tissues could potentially be regenerated by transplanting bioengineered cardiac patches to the heart surface. To be fully paradigm-shifting, such patches may need to be transplanted using minimally invasive robotic cardiac surgery (not only traditional open surgery). Here, we present novel robotic designs, initial prototyping and a new surgical operation for instruments to transplant patches *via* robotic minimally invasive heart surgery.

**Methods:** Robotic surgical instruments and automated control systems were designed, tested with simulation software and prototyped. Surgical proof-of-concept testing was performed on a pig cadaver.

**Results:** Three robotic instrument designs were developed. The first (called “Claw” for the claw-like patch holder at the tip) operates on a rack and pinion mechanism. The second design (“Shell-Beak”) uses adjustable folding plates and rods with a bevel gear mechanism. The third (“HeartStamp”) utilizes a stamp platform protruding through an adjustable ring. For the HeartStamp, rods run through a cylindrical structure designed to fit a uniportal Video-Assisted Thorascopic Surgery (VATS) surgical port. Designed to work with or without a sterile sheath, the patch is pushed out by the stamp platform as it protrudes. Two instrument robotic control systems were designed, simulated *in silico* and one of these underwent early ‘sizing and learning’ prototyping as a proof-of-concept. To reflect real surgical conditions, surgery was run “live” and reported exactly (as-it-happened). We successfully picked up, transferred and released a patch onto the heart using the HeartStamp in a pig cadaver model.

**Conclusion:** These world-first designs, early prototypes and a novel surgical operation pave the way for robotic instruments for automated keyhole patch transplantation to the heart. Our novel approach is presented for others to build upon free from restrictions or cost—potentially a significant moment in myocardial regeneration surgery which may open a therapeutic avenue for patients unfit for traditional open surgery.

## Introduction

Advances in regenerative medicine have raised the hope of restoring damaged heart muscle (Roche et al., 2020). Approaches include bioengineering patches for transplantation to the heart surface, which may utilize increasingly accessible methods such as 3D bioprinting (Roche et al., 2020; Roche et al., 2021a). Transplantation of patches containing cells and biomaterials to regenerate heart muscle (myocardium) has been performed in animal models (Gao et al., 2018; Roche and Gentile, 2020; Wang et al., 2021) and human trials (Chachques et al., 2007; Sawa et al., 2012; Menasché et al., 2015; Sawa et al., 2015; Menasché et al., 2018). Until recently (Roche et al., 2021b; Wang et al., 2021), open surgical approaches have been investigated (Wang et al., 2020), even though this could exclude heart failure patients unfit for the physiological demands of open chest surgery (but who might be fit enough to undergo a less invasive procedure). Furthermore, if patch-based myocardial repair is to realize its paradigm-shifting therapeutic potential (for example, as a standalone procedure and/or an adjunct for a patient already undergoing surgery for some other reason), it should be compatible with minimally invasive (keyhole) robotic surgical approaches (Roche et al., 2021b). Otherwise, it could be hard to justify a traditional open surgical operation, not least because the risk/benefit threshold may be harder to meet (Trevis et al., 2020). For example, conventional open surgeries, splitting the sternum (median sternotomy) or via the ribcage (thoracotomy), have been associated with a longer initial recovery and higher incidence of overall complications/adverse events than minimally invasive approaches (Lim et al., 2021; Moscarelli et al., 2021).

With the advancement of minimally invasive robotic cardiothoracic surgery (Torregrossa and Balkhy, 2020; Güllü et al., 2021), it is increasingly pertinent to develop feasible, cost-effective, synergistic instrument-control systems (Trevis et al., 2020). Current surgical robots established in clinical use, including da Vinci^®^ Surgical Systems (Intuitive Surgical Inc., Sunnyvale, CA, United States), have shown that a master-slave (semi-automated) robotic system can perform robotic surgery on humans, resulting in new ways to perform challenging cardiac surgery (Güllü et al., 2021) and potentially improving established operations (Torregrossa and Balkhy, 2020). However, robots like the da Vinci are currently expensive—one Sydney hospital recently reported the implementation cost alone for the *da Vinci Xi* was $4.4 million AUD (approximately $3.2 million USD) (McBride et al., 2021)—which may limit widespread implementation (Crew, 2020).

Here, we hypothesize that a feasible minimally invasive robotic approach for transplantation of cardiac patches could be developed without prohibitive costs. To achieve robotic keyhole cardiac patch transplantation, surgical instrument development was accompanied by development of customized control systems. We present world-first cardiac patch transplantation instrument designs, with simulated robotic control systems, along with initial prototyping and a proof-of-concept surgical test in a pig cadaver.

## Materials and Methods

Full methodological technical details are available in the Supplementary Materials and the complete reproducible dataset is freely available from the permanent data repository associated with this manuscript (https://doi.org/10.5281/zenodo.4784952). Supplementary Material was uploaded for peer review with the manuscript.

*Instrument Design Process and Objective Setting.* Three different instruments and two control systems for instruments were created using computer-aided design (CAD) software (SolidWorks, Waltham, MA, United States). Initial instrument designs, named “Claw”, “Shell-Beak” and “HeartStamp” after their mechanisms (which resembled, respectively, a claw, a bird’s beak closing to form a shell-like compartment, and a stamp for pressing patches onto the heart) were created based on learning from 12 months of preliminary design and resin-based part prototyping (Roche et al., 2021b). Instrument designs had to be able to transplant a hydrogel-based patch via minimally invasive heart surgery and to be capable of robotic control (for master-slave control or full automation). Additionally, designs had to be: 1) safe (made from biocompatible, non-toxic materials without sharp edges); 2) either disposable or sterilisable by autoclaving and/or sterile sheath covering during surgery; 3) the correct size for human use; 4) strong enough to withstand moment forces at the keyhole entry point (between the ribs at the chest wall); 5) feasible for prototyping without needing specialist manufacturing techniques and at a reasonable cost. Additional design objectives were added for specific instruments. For the Claw (2 cm diameter), it was designed to deliver multiple small patches to precise areas of the heart surface one after the other. For the Shell-Beak (2 cm diameter) the objective was to deploy a cardiac patch which is larger than the diameter of the cardiac instrument. For the HeartStamp (5 cm diameter) additional objectives included: 1) sizing—the diameter of the prototype should be 5 cm and the length greater than 30 cm; 2) ease of use—the design must be intuitive enough for a surgeon to be able to use the device with no additional training; and 3) a surgical camera as well as keyhole forceps must be able to fit inside the device to ensure the surgery is minimally invasive. Ideas were formalized using Scamper tables, tree diagrams, CAD and materials lists for prototyping (see the Supplementary Materials)**.** The full methodological datasets **(**including SolidWorks files and components) are available in the permanent data repository (https://doi.org/10.5281/zenodo.4784952).

*Surgical Compatibility.* The Claw and Shell-Beak were designed to fit 2 cm diameter keyhole ports in the chest wall (a frequently used diameter for multiple port keyhole surgery). The HeartStamp was designed for a specific cardiothoracic surgical approach: uniportal Video-Assisted Thorascopic Surgery (VATS)—an approach where all keyhole instruments are inserted via one slightly larger (∼5 cm) port used in thoracic surgery. The HeartStamp was also designed to be prototyped from readily available materials and compatible with autoclaving and/or sterile sheath covering.

*Proof-of-concept Control System Designs and Simulations (in silico).* Using SolidWorks and TinkerCAD (San Francisco, CA, United States), two instrument control systems were designed and simulated. Control System 1 was designed for (and applied to) the Claw design instrument to show compatibility with one of the instrument designs (*in silico*). Control System 2 was designed to control nine rods in a 2 cm diameter (keyhole) cylinder, aimed at demonstrating individual rod control for up to nine rods and multiple (greater than 6) degrees of freedom for instrument movements—tailored to our previous preliminary instrument design (Roche et al., 2021b). Systems used either screw-driven rotationary force or push-pull and gear-based mechanisms to control instrument movements and release the patch. Technical details are available in the permanent data repository (https://doi.org/10.5281/zenodo.4784952).

*Proof-of-concept 3D Print Prototyping (Control System 2).* Control System 2 was prototyped to show proof-of-concept. A Polylactic Acid (PLA) “sizing and learning” prototype was 3D printed (layer resolution 100 μm, PLA filament diameter 1.75 mm, nozzle diameter 0.4 mm) using a MakerBot Replicator+ extrusion 3D printer (MakerBot, New York, NY, United States) followed by manual assembly of parts to show proof-of-concept feasibility. The complete Makerbot STL file instructions sent to the 3D printer are available in the permanent data repository (https://doi.org/10.5281/zenodo.4784952).

*Proof-of-concept Prototyping (HeartStamp Instrument).* To determine the optimal prototyping method according to our objectives, we engaged in repeated brainstorming sessions and further developed those ideas using the tables, charts and CAD files shown in the Supplementary Materials. We purchased basic hardware materials (for the complete list with catalogue numbers, supplier and pricing see dataset at permanent https://doi.org/10.5281/zenodo.4784952) before manually self-assembling them to create the prototype.

*Proof-of-concept Surgery.* One researcher (CDR) performed the surgery (with the assistance of CG) using standard surgical materials (including a Haight-Finochietto rib retractor, Symmetry Surgical, Nashville TN, United States) and the HeartStamp prototype. A pig cadaver model was used (an anatomically suitable and ethically permissable model chosen because it could fulfil requirements for proof-of-concept testing without any animals being harmed). We used a combination of moulded and 3D bioprinted hydrogel patches—alginate 4% (w/v) / gelatin 8% (w/v) in Dulbecco’s Modified Eagle Medium, previously optimized for cardiac applications (Roche and Gentile, 2020; Roche et al., 2021a). All patches were generated the day before the test and stored at 4°C overnight. On arrival, the pig cadaver was laid in the right lateral position, surgical field prepared with drapes and incision sites marked. To maximize utility, two surgical incisions/approaches were attempted one after the other: 1) left antero-lateral (left of the midline from the front of the chest to the side) and 2) left postero-inferolateral (left of the midline, towards the back, lower down and to the side). The anterolateral approach aimed to simulate a minimally invasive (trans)apical approach (left 5th intercostal space, mid-clavicular line) and the postero-inferolateral approach aimed to simulate a type of approach suitable for uniportal VATS. To simulate real surgical conditions, the operations were performed in real time with one attempt each and reported in full. Operations were video recorded/photographed and observed by an independent observer (Technical Operations Manager, Sydney School of Veterinary Sciences) who assisted when required (including abducting the right upper limb so it did not obstruct the surgical field). The operating room ambient temperature was 4°C and the HeartStamp was operated manually by the surgeon with all mechanisms tested by movements which would be feasible when connected to a robotic control system.

## Results

### Early-Stage Instrument Design Outcomes

**FIGURE 1:**
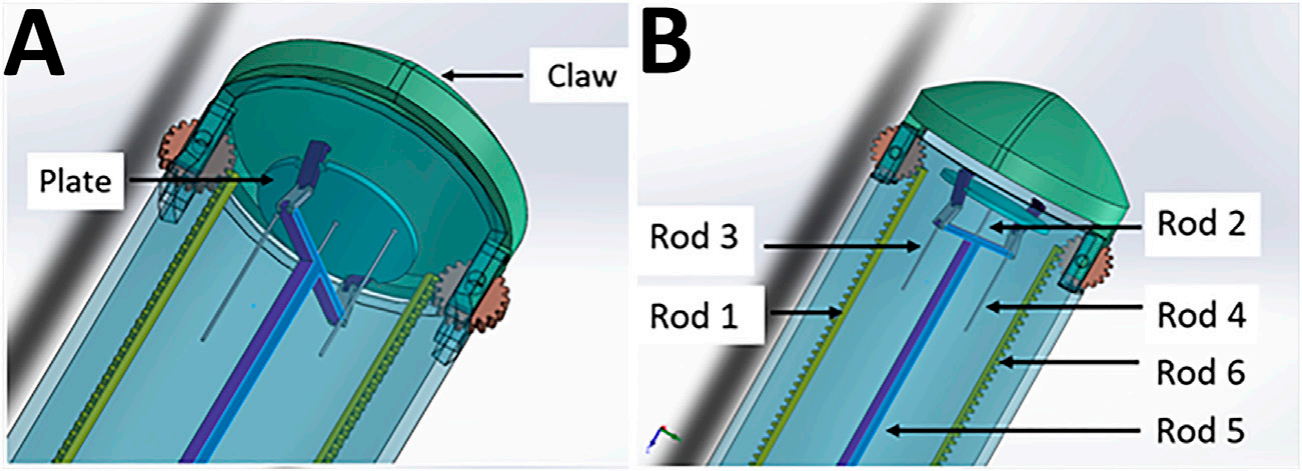
The Claw design in detail. **(A)** The plate (transplantation platform) where the hydrogel patch sits, enclosed by the “claw” to create a safe compartment. **(B)** Controller rods allow opening of the claw (Rod 1 and 6), changing the phasing angle of the plate (Rod 3 and 4), release of the patch securing clips (Rod 5) and protrusion and retraction of the platform (Rod 5 supported by Rod 2). For clarity, Rods 3 and 4 are shown cut short but would actually travel through the instrument to the proximal (control unit) end with the other rods (see also Figure 6C which shows the whole length and proximal connections of rods to micro-servo motors).

**FIGURE 2:**
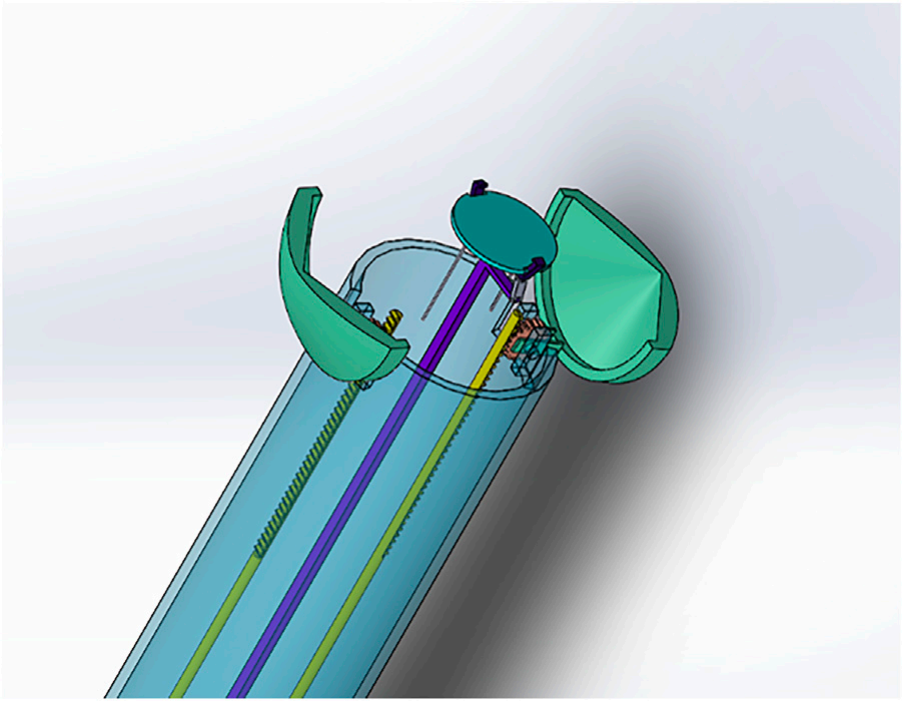
Claw design (open position with plate holder protruding). For secure robotically-enabled transplantation of cardiac patches using minimally invasive heart surgery.

*Claw Design.* The Claw instrument was designed to maintain narrowness of diameter while enclosing a patch in a safe compartment at the distal tip, which would allow the surgeon to manipulate it with no risk of the patch falling off before opening over the target area. The designs achieved a 2 cm diameter while also holding a patch on the transplant platform until the moment of release (Figure 1, Supplementary Figure S1 and Supplementary Video S1). To avoid the patch falling or being knocked off the instrument during transplantation to the heart, the head was designed with two claws in the shape of a rounded-base triangle on a cylinder arc or a tapering rectangle with the short edges meeting together (initial designs shown in Supplementary Figure S2). The initial designs did not completely enclose the patch compartment, therefore in the final design the infolding panels forming the “claw” part were changed to a cone shape on a circular base (20 mm base diameter and 19.88 mm overall height—complete measurements shown in Supplementary Figure S1). The infolding-outfolding mechanism used a ball joint with a rack and pinion (Supplementary Figure S3 and Supplementary Videos S1, S2). The resulting final Claw design (shown in complete form in Figure 2 and Supplementary Video S1) consists of two “claws” at one end (responsible for opening and closing of the instrument) and a plate holder (Supplementary Figure S4) with clips to secure the patch during shift in phasing angle (enabling control of the precise position of patch delivery). The plate holder was designed with a diameter of 9.80 mm considering the keyhole surgery incision (Supplementary Figure S4).

As observed in Figure 1, Controller Rods 1 and 6 are responsible for controlling the open and close action for the “claw” component of the instrument holding the patch in place. Both the rods work on a rack and pinion system with a gear of 20 teeth (Supplementary Figure S3 and Supplementary Video S2). The gear is in turn connected with the claws, thus allowing movement for opening and closing the instrument (Supplementary Video S1). Once the claws are open, Rod 2 facilitates protrusion of the plate holding the patch (the transplantation platform) out of the instrument. Cardiac patches will be placed on the plate and held in position by the clips connected through Rod 5. After the plate is pushed out of the instrument to the desired position, Rods 3 and 4 will be moved in a linear position to change the phasing angle of the plate. This is done to achieve a precise angle to position the patch on the heart (Supplementary Video S1). This is facilitated by three joint mechanisms—which we have labelled as *L-link* connected to the plate from one end and attached with *T-link* from the other end through *H-link* (as shown in Supplementary Figure S5). Once the position of attachment for the cardiac patch is determined, Rod 5 will move in a linear direction to release open the clip thus releasing the patch from the instrument (*via* a ball joint). Using Rod 2 for support, the patch can be pressed on the required target site (Supplementary Video S1).

**FIGURE 3:**
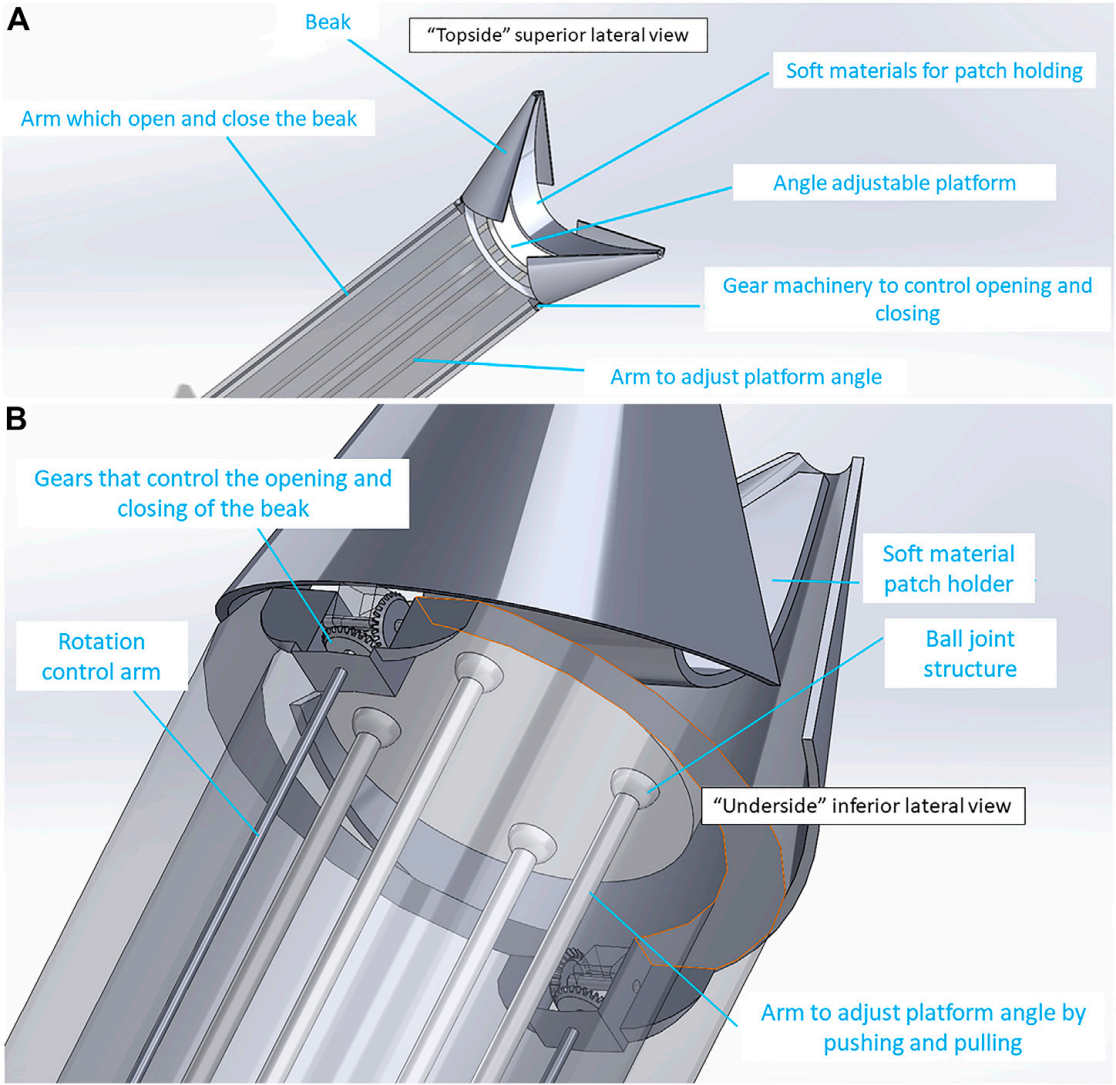
“Shell-Beak” design with flexible patch holder connected to the patch dispenser system. **(A)** “Topside” superior lateral view of the distal (head) of the instrument with partially open beak and flexible patch-holder with space for an infolded patch between the patch-holder and the adjustable platform. **(B)** “Underside” inferior lateral view looking at the base of the patch platform. Ball joints and gear mechanism are shown which allow for changes to the phasing angle of the platform and opening/closing of the beak, respectively.

*Shell-Beak Design.* To address the potential challenge of transplanting a patch larger than the instrument platform, we designed a second instrument called “Shell-Beak” (Figure 3). The design was inspired by the functionality of a bird’s beak and simplicity was sought to maximize feasibility. Conceptually, the distal end of the instrument holds an infolded strip of patch, which can be angled during transplantation and pushed out towards the target site. The motion forces would be transmitted with simple push/pull movements of rods surrounded by an encasing cylinder. The patch dispenser has a coin-like base at both ends, in which the patch could have one attachment when infolded to the distal base at the releasing end of the instrument (Supplementary Figure S6). The rods and base are connected using ball joints, which gives flexibility to the structure so that is can hold the patch, adjust its facing angle, and push it towards the targeted site during operation. This mechanism is facilitated using a push/pull controller rod arrangement (Figure 3). The opening and closing of the beaks were designed using a bevel-gear system. This mechanism converts rotational motion to rotational motion (unlike in the Claw design where linear motion is converted to rotational motion). The angle adjustment platform is responsible for controlling the phasing angle and patch positioning onto the target site. The rotational control of the arm is designed to activate the bevel-gear mechanism to open and close the beak head. An inwardly concave patch holder was conceptualized (Figure 3), which would be fabricated from a soft and flexible material and this would sit on top of the patch (with the patch sandwiched between the holder and the distal coin base). The tips of the patch holder would be linked to (or clipped into) the distal tips of the beak, so as to facilitate the instrument holding a patch of eliptical shape of up to ∼10 cm in length.

**FIGURE 4:**
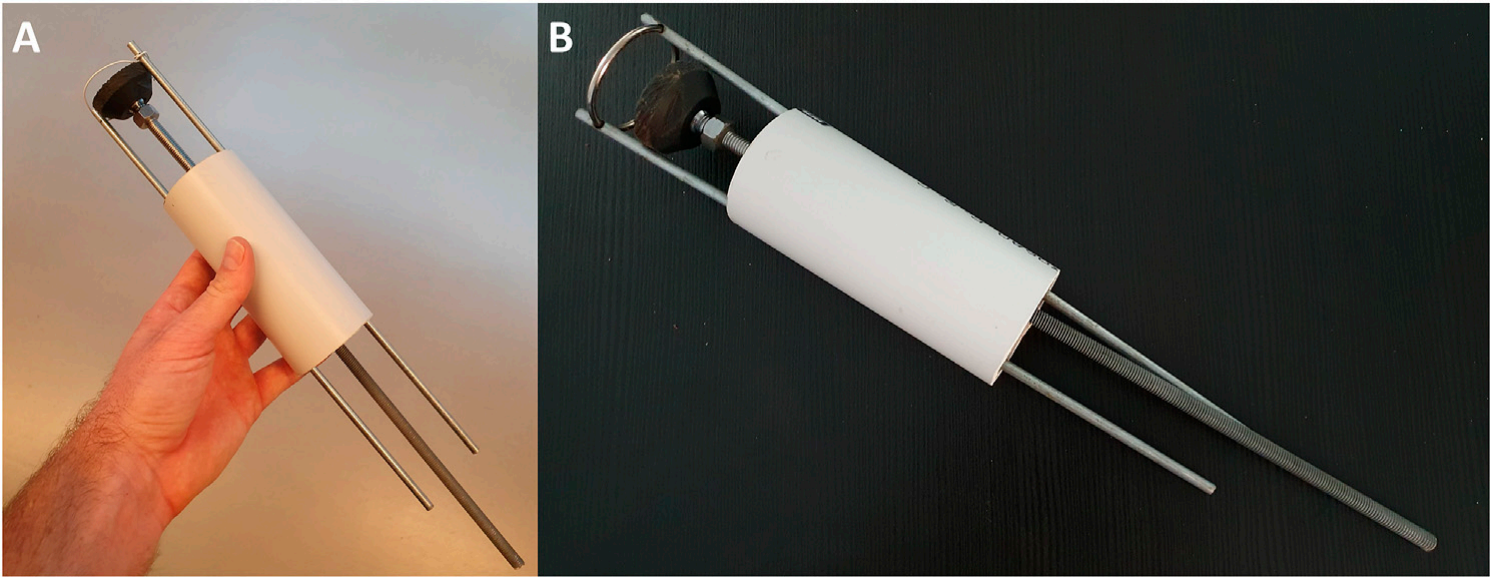
The HeartStamp prototype for cardiac patch transplantation. The HeartStamp is designed to transplant a patch to the surface of the heart via a uniportal VATS incision. The instrument is shown prior to use at surgery with a flexible ring and the operator’s hand for scale **(A)** and with a rigid ring after use at surgery **(B)**. Computer-aided design (CAD) images associated with this prototype are shown in Figure 5.

**FIGURE 5:**
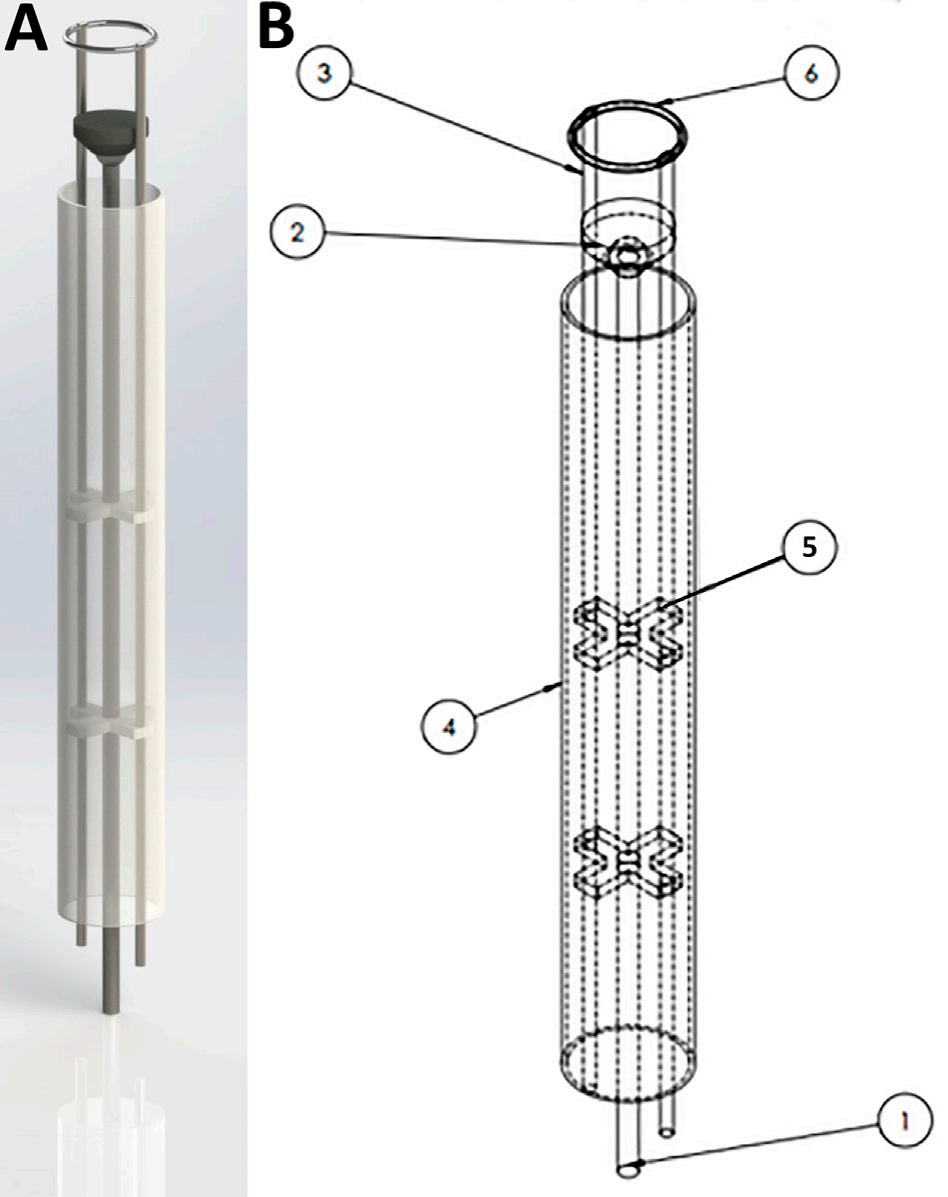
SolidWorks design for the HeartStamp device prototype. 3D rendering of the HeartStamp instrument **(A)** and technical details with labelled parts **(B)**. Item labels: 1) Central Rod (controls the puck); 2) Puck (pushes patch off the ring and onto the heart); 3) Paired Ring Rod (controls the ring angle); 4) Tube (provides protection for components-tube length is modifiable with a long tube shown here whereas a short tube version was used at surgery and shown in Figure 4); 5) Paired X-shaped Support (supports the rods); 6) Metal ring (the heart patch is rested on this). The resulting prototype generated from these computer-aided designs is shown in Figure 4 and the raw data are available in the Supplementary Materials.

*HeartStamp Design.* This design (Figures 4, 5) was created to be readily prototyped from standard materials while still satisfying the requirements for robotic minimally invasive patch transplantation. It was designed to fit a control unit with: 1) a central rod protruding and retracting a “stamp”; and 2) two lateral rods attached to a ring which could change the phasing angle of the ring (if one is activated) or extend the longitudinal range of the instrument without changing the ring angle (if both are activated equally). The HeartStamp is a second-generation design and prototype based on a previous design concept called “Umbrella” (Supplementary Figure S7) and incorporating elements of the Claw and Shell-Beak. The first-generation (Umbrella) design included a partially-flexible cylinder accommodating independent wires and several joint spaces. A central push platform was used to push the patch onto the heart. For the HeartStamp (Figures 4, 5), the design was optimized for surgery so that the central push mechanism was surrounded by a rigid cylinder able to sit between the ribs in a uniportal VATS entry hole in the chest wall (with or without the use of a retractor to spread the ribs). The push stamp platform (puck) is attached to a central rod travelling through the cylinder to the control unit (or surgeon’s hand if being deployed manually). The stamp platform is surrounded by a metal ring with rods at the 12 o’clock and 6 o’clock positions). Both rods travel through the cylinder and can be protruded and retracted by the control unit, allowing for the phasing angle of the ring to be adjusted. The device is autoclavable and/or compatible with a sterile sheath. If a sheath is used, the stamp platform contacts the sheath and pushes it out, pushing the patch off from its position on the plastic sheath on the opposite side*.*

### Control Systems 1 and 2

We designed two control systems/units and ran *in silico* simulations using CAD software as described below.

**FIGURE 6:**
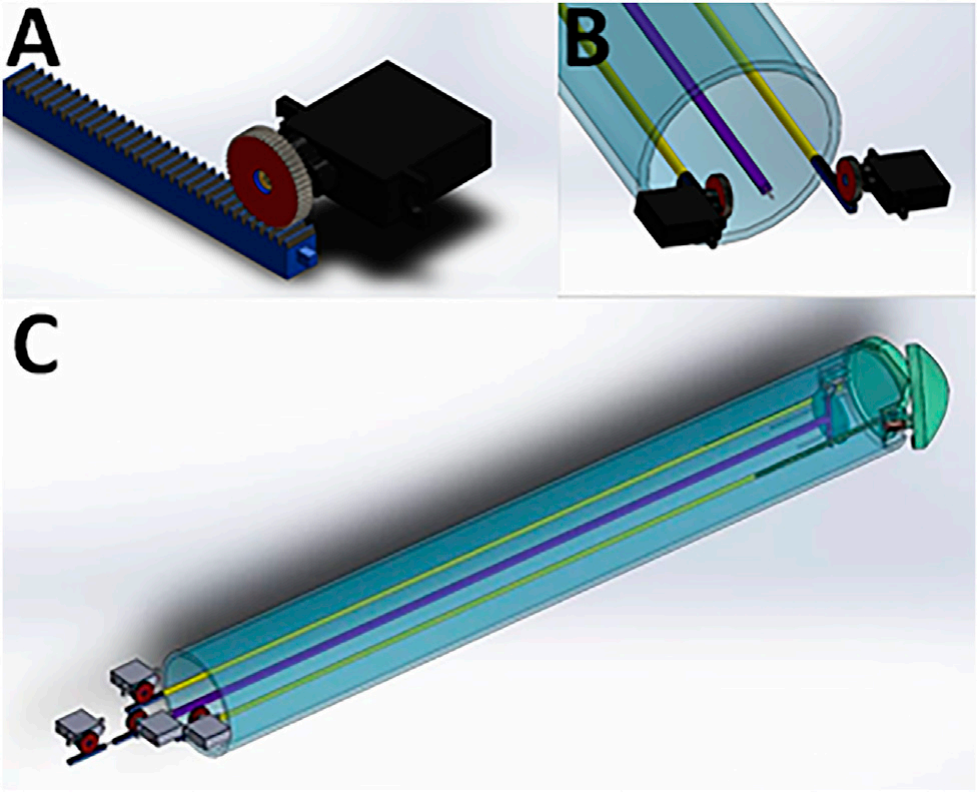
Mounting of micro-servo motors onto the rods of the instrument using the Claw design as an example. **(A)** One micro-servo motor unit attached to a rod. **(B)** The proximal (tail end) of the instrument showing two micro-servo motor units. **(C)** View of the whole instrument, showing four micro-servo motor units and the rods connecting the proximal to the distal end.

*Control System 1.* To control the Claw, a control system with an Arduino microcontroller and micro-servo motors was designed (Control System 1, Figure 6 and Supplementary Video S2). This was simulated *in silico* (Supplementary Video S2), suggesting that the surgeon would be able to control the instrument using a computer console. The control system could be connected to the instrument head using mechanical components from the other end of the system (such as rods passing through a cylindrical tube). The overall dimension for this design was kept to 2 cm maximum diameter, meaning that the instrument could pass through small keyhole ports if a strong material and high-resolution manufacturing technique was used for the small parts. To demonstrate the working of the instrument for simulation purposes, an Arduino microcontroller was coded with micro-servo motors connected to each rod (Supplementary Video S2) - simulating *in silico* the working of a linear actuator using micro-servo motors. TinkerCAD was used for this simulation purpose. The connections are outlined in Supplementary Video S2. Each micro-servo motor is connected to a potentiometer. The potentiometer acts like a common “regulator” which when rotated in a clockwise direction will move the micro-servo motor, which in turn will result in an individual rod within the instrument moving in linear motion. The control system will be mounted onto the rods as shown in Figure 6 and Supplementary Video S2. On rotating the potentiometer in a clockwise direction, this will result in the rod moving in the forward direction. Similarly, when rotated in an anti-clockwise direction the rod will be retracted. If applied to the Claw design instrument, for example, Potentiometer 1 can be connected to the two micro-servo motors opening the Claw (Figure 6 **)**, wherein both Rod 1 and Rod 6 will be positioned in a mirror manner. Potentiometer 2 controls Rod 2 to take the cardiac patch securely outside the device. This rod can then be controlled to 360 degrees in any direction to position the patch over the target site. Similarly, Potentiometers 3 and 4 control Rods 3 and 4, respectively, to change the phasing angle of the plate over which the patch sits. Rod 5 controls the releasing of the clips over the plate that secure the patch onto the plate. Linear movement of Rod 5 results in opening and closing of the clip thus leading to release of a cardiac patch on the target site. The potentiometer acts as a joystick with which the surgeon can alter the linear motion of the rods connected to the micro-servo motors. Overall, this approach presents a control system to control the push and pull motion for each rod of an instrument with a similar mechanism to the Claw.

**FIGURE 7:**
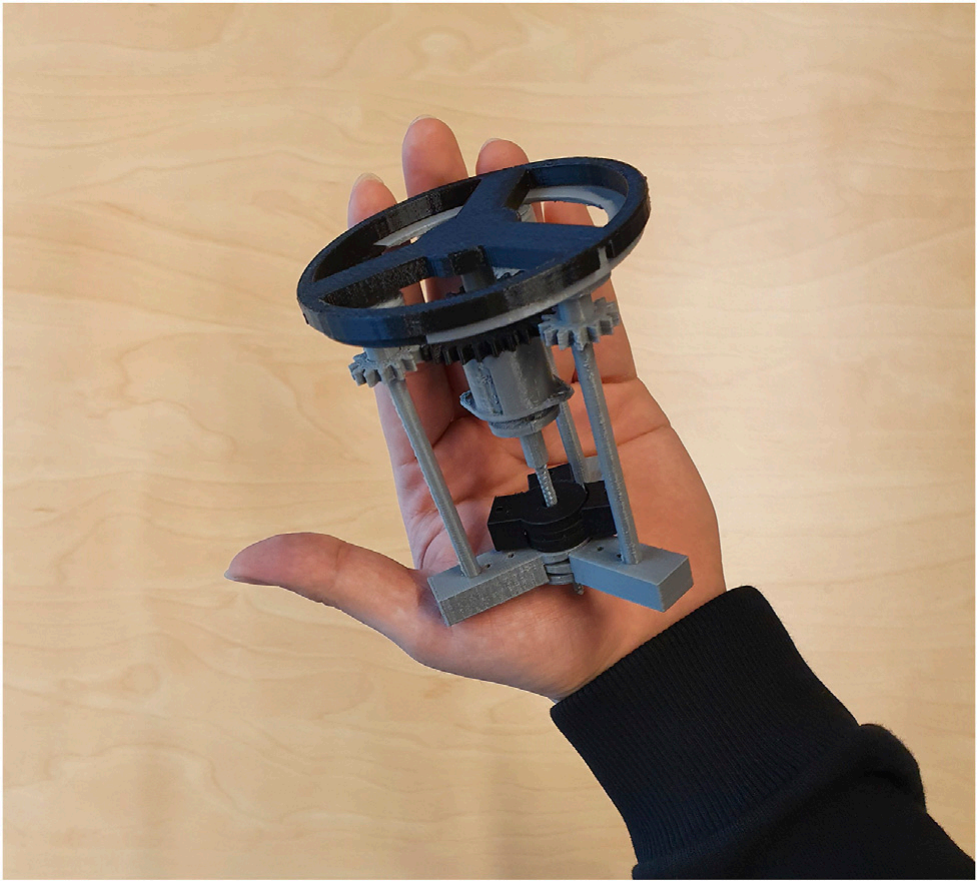
A proposed control unit sizing and learning polylactic acid (PLA) prototype. This control unit was prototyped to show preliminary feasibility and identify learning points in preparation for more extensive metal prototyping. It is the complete triplicate prototype for the single subunit (one third) mechanism shown in Supplementary Video S3. Each subunit controls three rods and the three subunits are added together in a wheel-and-spoke fashion with 120 degrees between each subunit. The complete control unit was designed to control up to nine rods in a narrow-diameter (2 cm) keyhole surgical instrument such as the one detailed in our preliminary brief research report (Roche et al., 2021b).

*Control System 2.* To control more rods, we designed a second control unit based on a different approach capable of controlling nine rods at the same time (Control System 2, Supplementary Video S3)—tailored to an early instrument design invented during our previous preliminary work (Roche et al., 2021b). This was designed to provide more degrees of freedom (planes of movement) in a low-diameter (2 cm) keyhole surgical instrument. Thanks to our *in silico* simulation, we tested its rod retraction, protrusion and rotation, where push-pull forces could be applied to nine rods via three U-shaped subunits/connectors (Supplementary Video S3). Each subunit connector was designed to move each rod independently and to combine movements to move either two or three rods simultaneously. This subunit is replicated three times in a wheel formation with 120 degrees between each of the three subunits (connected to three rods each) in the resting position. A prototype for this control unit was 3D printed followed by manual assembly of parts to show proof-of-concept feasibility (Figure 7).

### Proof-of-concept Surgery (Pig Cadaver)

To show surgical feasibility of our approach, we operated on a porcine cadaver for the transplantation of patches using the HeartStamp. The pig had died of unrelated causes and its cadaver was provided by the University of Sydney Veterinary School (which avoided disposal without utilisation and avoided use of a live animal for testing). To simulate real surgery, each of our surgical approaches was performed once in real time and reported in full.

**FIGURE 8:**
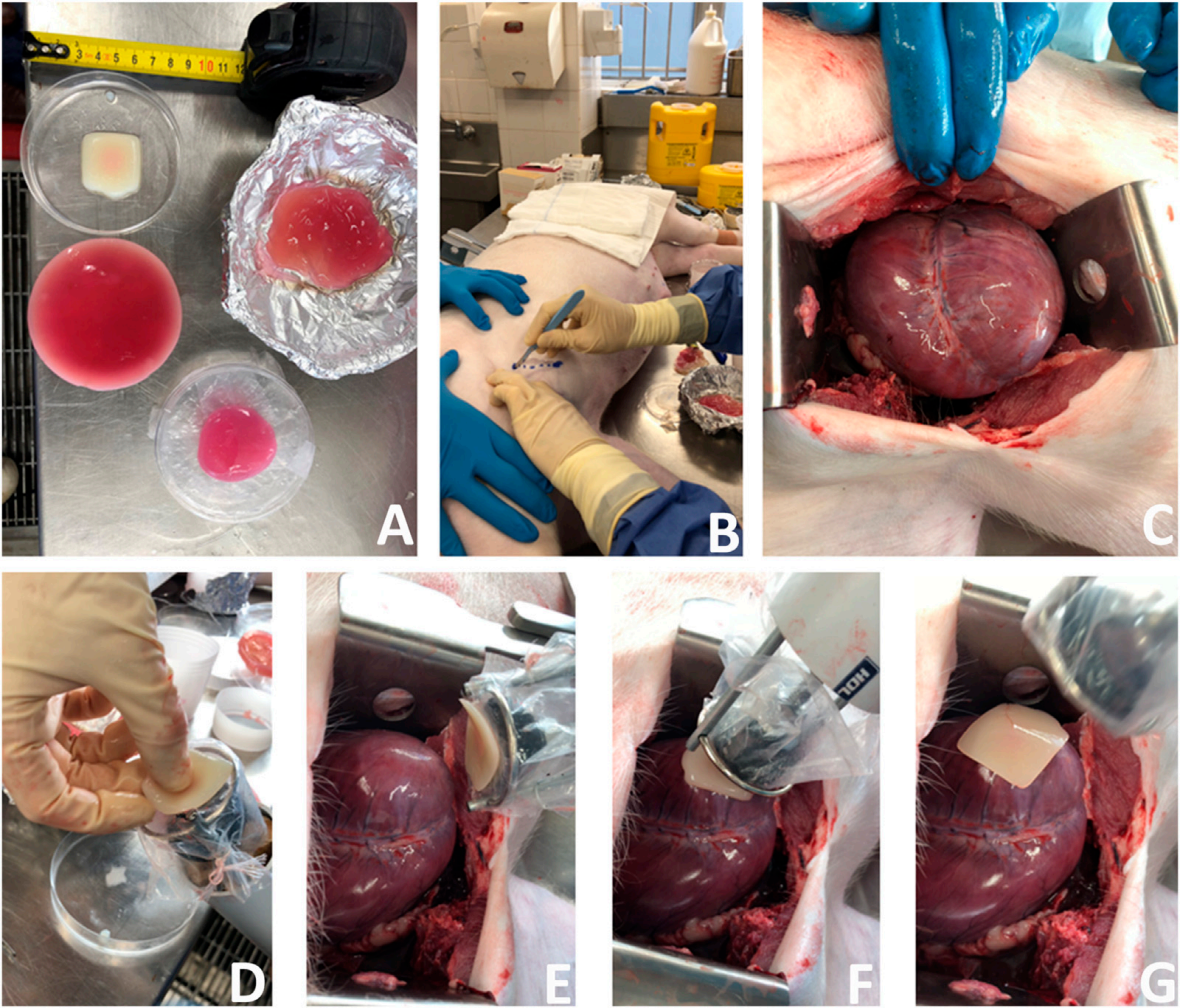
A new surgical operation for epicardial patch transplantation using the HeartStamp—left anterolateral approach with sheath. This surgery was performed once in real time, accepting complications to more closely simulate real surgical conditions (because the heart was enlarged and not accessible using the usual anatomical landmark we had to convert this operation to an open (not keyhole) approach—a complication which happens in human cardiothoracic surgery as well). We chose to continue and report the operation exactly as it happened. **(A)** Patches for cardiac applications were created using alginate/gelatin hydrogel by 3D bioprinting (the white patch) or moulding techniques (pink patches). **(B)** A left anterolateral surgical incision was made in a fresh cadaver of a pig which had recently died of unrelated causes. **(C)** The incision was extended, the chest wall tissues retracted, pericardium cut to expose the heart and the heart manouvred slightly into position. **(D)** The 3D bioprinted patch was manually applied to the plastic sheath at the distal (patient) end of the HeartStamp instrument. **(E)** The HeartStamp was moved into position by the surgeon and the patch remained lightly adherent to the plastic sheath. **(F)** The patch was transplanted to the epicardial surface over the left ventricle. **(G)** The HeartStamp is withdrawn, leaving the patch *in situ* on the epicardium. To complete the operation, the patch was then secured under the pericardium and the chest closed surgically.

*Surgical Approach 1.* We initially tried a minimally invasive left antero-lateral (left of the midline from the front of the chest to the side) approach (Figure 8) to mimic that used for access to the heart tip (apex). However, the pig heart was enlarged (due to previously established heart failure) and the incision in the usual anatomical landmark of the 5th intercostal space was too high to visualize the cardiac apex and therefore the incision had to be converted to a larger, open surgical approach (a complication in human cardiothoracic surgery as well). Nonetheless, using this approach we were able to visualize the anterior interventricular (left anterior descending) artery and transplant a patch to the often-infarcted territory supplied by this artery (Figure 8). We used a plastic sheath to simulate a sterile surgical instrument sheath and placed a 3D bioprinted alginate/gelatin patch onto the heart surface. The patch adhered to the sheath, even when turned vertically upside down (Figure 8; Supplementary Video S4). The stamp mechanism was used to push the patch off the plastic onto the heart by protrusion of the stamp (central rod) while stabilising the ring (lateral two rods). The stamp contacted the inner surface of the sheath, pushing it out and the patch was released from the outer surface.

**FIGURE 9:**
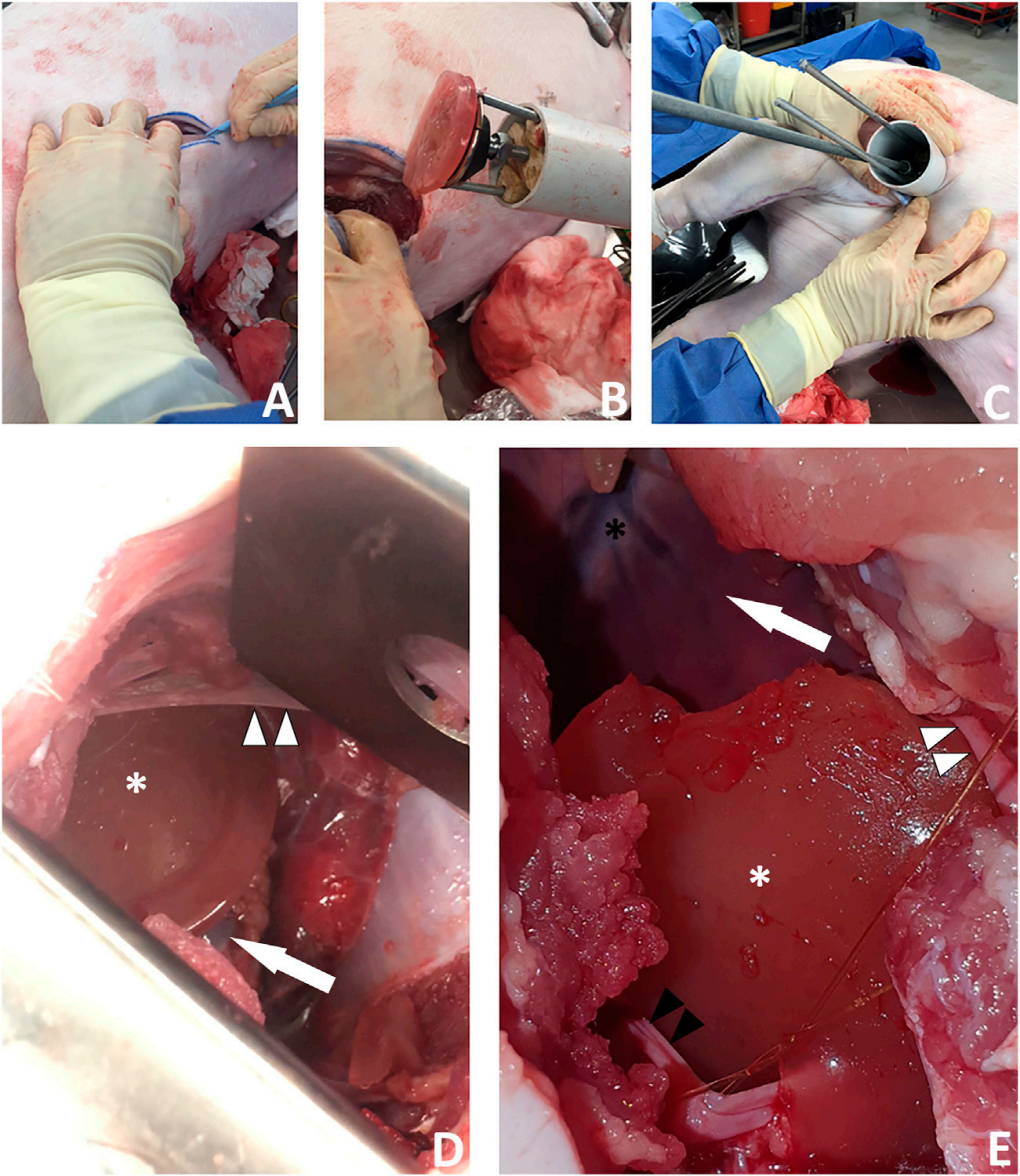
A new surgical operation for epicardial patch transplantation using the HeartStamp—left postero-inferolateral approach without sheath. This surgery was performed once in real time to better simulate real surgical conditions and there were no complications. **(A)** A postero-inferolateral surgical incision was made in a fresh cadaver of a pig which had recently died of unrelated causes. The previously made left antero-lateral incision (the other approach we tested shown in Figure 8) is concealed under the surgeon’s left hand in the photograph shown in panel **(A)**—higher up and more towards the midline. **(B)** A moulded alginate/gelatin hydrogel patch was picked up by the instrument (without manual application) and is shown here adherent to the ring at the distal (patient) end of the HeartStamp instrument. The black stamp head is seen in retracted position under the patch. The HeartStamp is moved into position by the surgeon. **(C)** The HeartStamp is shown in position, inserted through the incision in the chest wall (the 5 cm diameter HeartStamp cylindrical body fits the 5 cm incision). This entry route is similar (in location and size) to established approaches used for uniportal (one port for all surgical instruments) VATS—a type of keyhole surgery on the chest. The rods are seen from the control (operator) end with the two shorter rods being lateral and connected to the ring (distal end—inside the patient) and the longer rod being central and connected to the stamp. **(D)** For confirmation that the patch was successfully transplanted (as shown in Supplementary Video S4), we used a retractor to open the wound and visualize the patch (white asterisk) on the apex of the heart (white arrow). A thin beam of pericardium is seen superiorly (white arrowheads). **(E)** Close-up view of the patch (white asterisk) shown on the heart apex (not shown—concealed by the patch) after we opened the chest fully to examine the patch location. Left/superior pericardial tissue is seen (white arrowheads) and right/inferior pericardium (black arrowheads) is visualized attached to a surgical suture. The heart is seen in the background of the image (white arrow) along with the anterior interventricular (left anterior descending) blood vessels (black asterisk).

*Surgical Approach 2.* We made a 6 cm incision aiming for the porcine left 6th intercostal space, posterior axillary line. We cut through the soft tissues, separated the ribs with a retractor and cut the pericardium over the apex of the heart. We then removed the retractor and used the HeartStamp without a sheath to directly pick up a patch on the operating table (Supplementary Video S4). The patch was adherent to the ring and the two protruding ends of the lateral rods which control the ring. The instrument was inserted manually into the chest and operated manually by engaging the stamp protrusion mechanism. The stamp moved forwards and pressed the patch onto the heart surface at the apex (Figure 9 and Supplementary Video S4). We checked the position of the patch afterwards and confirmed it was *in-situ* at its intended location. We then closed the surgical wound, suturing the pericardium back together on top of the patch to secure it in place. All the movements (patch pick-up, patch transfer and patch release onto the heart) were enacted manually by the surgeon and were consistent with those possible using robotic control mechanisms.

## Discussion

We have demonstrated early-stage proof-of-concept feasibility for patch transplantation via robotic minimally invasive approaches. We have presented our designs in detail (including those which were not taken forward to prototyping) because each design may have a specific value for certain types of patch. Considerations for the patches themselves are described in detail elsewhere (Roche et al., 2021a), but for example, alginate/gelatin (Roche and Gentile, 2020), fibrin-based (Menasché et al., 2018) or more complex matrices such as methacrylated elastin/gelatin/carbon-nanotube would all have very different properties/shape-memory behaviours (Wang et al., 2021). We have made all of our early-stage proof-of-concept data freely available for others to build upon and further trials will be required (including optimising instruments for certain patch types) for full systematic efficacy analyses. Nonetheless, our proof-of-concept surgical test of one of our prototyped designs (the HeartStamp) represents a world-first and potentially a significant step forward. The novel work presented in this article is the result of a collaborative study bringing together experts in robotics, mechanical engineering, cardiothoracic surgery and biofabrication. We are the first to present both the approach (epicardial heart patch transplantation by robotic minimally invasive cardiothoracic surgery) and instruments (designs and initial prototyping) to achieve this goal.

Our approach overcomes several mechanistic limitations which occurred in our design process. This is illustrated by our ‘Claw’ and ‘Shell-Beak’ designs which had a 2 cm diameter to make them compatible with conventional keyhole surgery (that is, three or more 2 cm ports and the surgeon triangulating) as well as uniportal (all instruments in one 5–6 cm hole). For example, with the Claw design we initially encountered the problem that the facing angle might be nonadjustable, meaning it would have to be inserted perpendicular to the contour of the target heart surface (Supplementary Video S1 **).** To overcome this, we used a rack and pinion system combined with ball joints which allowed changes to the phasing angle (Supplementary Video S1). With this design (Figures 2, 6), the Claw releasing compartment was supported by a combination of linkages and ball joints and the phasing angle could be adjusted to align with the contact contour of the target area. Conversely, for the HeartStamp (Figures 4, 5), we realized that a clamping method to hold down the patch was not needed due to the sticky nature of our alginate/gelatin patches (Supplementary Video S4). This finding allowed us to keep a simple design whereby a patch could be pressed on the heart in the desired location (similar to a stamp, a puck was pushed out through a ring to detach the patch off the ring and onto the heart). In this case, the phasing angle could simply be controlled by two independent rods controlling a ring through which the puck presses and this mechanism worked both when covered by a plastic sheath and without a sheath. It remains an open question whether the stickiness of the patch itself could be controlled to avoid adhesions and deformation, and this is especially important for any infold-outfold designs such as the Shell-Beak (Figure 3). The flexible patch holder elipse of the Shell-Beak (conceptualized to sit on top of the patch) could potentially assist against patch deformation. Such a patch holder could potentially even be generated at the same time as the patch and used as a patch loading and stabilising mechanism (although this would need further testing and questions would remain such as where the patch holder goes at the moment of transplant). Due to the stickiness of the alginate/gelatin patches, there should be no requirement for a forceful releasing mechanism with any of our systems, provided the plate is made from non-adhesive material (less adhesive to the patch than the heart is). It has been shown that hydrogels can remain in situ in the sub-pericardial space (Zhu et al., 2021) and our patches (which do not retain sutures) are also designed to be secured under the pericardium.

As instruments without control mechanisms would have limited usefulness, we developed control systems to operate instruments robotically. For any control system, at the operator’s end (the tail of the instrument), it is crucial to consider appropriate connections of the instrument to the control system. Our Control System 1 demonstrated the working of an instrument for simulation purposes *via* an Arduino microcontroller which was coded with micro-servo motors connected to each rod (Supplementary Video S2). The objective was to replicate *in silico* the working of a linear actuator using micro-servo motors (the connections are outlined in Supplementary Video S2 **).** While connecting multiple micro-servo motors with Arduino microcontrollers may seem uncomplicated *in silico*, there are potential limitations for its translation to the clinic. For example, if we connect all the servos to Arduino supply pins (outside a computer simulation setting) then they may not work optimally in standard operating theatres because of a lack of current to drive all the motors. When developing a real-time prototype, we would probably have to use a servo driver PCA 9685 and a relay. It should be noted that the Arduino is a first step and future work may include custom PCB boards, voltage rectifiers and/or a field-programmable gate array (FPGA). Nonetheless, in simulation, we successfully used micro-servo motors along with an Arduino UNO to demonstrate this feasible approach.

For Control System 2, our *in silico* proof-of-concept demonstration (Supplementary Video S3) and 3D printed prototype of the unit (Figure 7) paves the way for full functional prototyping and connection with a robotic mechanism to initiate and cease motion via a software controller. This control system design shows that it may be possible to control a large number of rods despite a tiny space, provided it is made from strong material using high-resolution manufacturing techniques. By altering the 120-degree angles of the spokes, rotation of groups of three adjacent rods at the same time is possible (Supplementary Video S3). At the distal face of the control unit (the patient-facing side which would be closest to the patient—where the rods exit the control unit), a gear mechanism is connected to the rods. This gear mechanism is designed to allow rotation of all the rods *en masse*. The combination of movements should allow for a large number (greater than 6) of degrees of freedom. This opens up the possibility of robotic initiation and maintenance of multiple movements simultaneously—well beyond the computational power of a human controlling the instrument manually.

The culminative achievements presented herein are the surgical patch transplantation operations (Figures 8, 9 and Supplementary Video S4). Of the two surgical approaches we tried, the more successful was *via* a minimally invasive approach using a left postero-inferolateral (left of the midline, towards the back, lower down and to the side) incision similar (in terms of size and entry point) to that which could be used for certain operations by uniportal VATS—where all keyhole instruments and the camera are inserted through one port (Figure 9 and Supplementary Video S4). We purposefully ran these operations once each in real time and we report them exactly as they happened, including any complications, to better simulate real surgical conditions. These proof-of-concept operations lay the foundations for more extensive trials, during which several limitations will need to be addressed. These include application of the instrument to a robotic control unit so that the manual operation component is removed, *in vivo* testing so that feasibility is demonstrated with a beating heart (although some cardiac surgery is performed on an arrested heart using cardiopulmonary bypass, this technique should be appropriate for beating heart surgery), performance of surgery in a theatre environment which is more closely controlled to resemble that of human surgery, optimising the exact surgical approach and quantifying complication rates over high numbers of repeats, as well as obtaining quantitative data for full analysis to show safety and efficacy.

Our overall objective was to develop a surgical robotic invention using accessible feasibility-focused approaches with low costs (for instance, the HeartStamp unit production cost was less than $100 AUD). Others have used a similar approach (avoiding prohibitively high costs) to develop master-slave systems working on the principle of semi-autonomous control (Bai et al., 2017; Zhou et al., 2018). Fully autonomous systems have also been developed, although human surgeon supervision may still be needed (Shademan et al., 2016). Fully autonomous unsupervised robotic systems for surgery would need to overcome safety concerns (Trevis et al., 2020). For example, it is foreseeable that a component or power malfunction (unsupervised) at a key moment in the surgery could lead to patient harm—after all, human cardiothoracic surgeons do not work alone unsupervised. Nonetheless, to future-proof instruments against technological progress making them redundant, they should ideally be compatible with both master-slave and full automation.

Overall, robotic-assisted keyhole surgery promises remarkable advantages for the cardiothoracic surgeon, especially if it can be done remotely (Torregrossa and Balkhy, 2020). Paradigm-shifting scenarios could be unlocked, such as for emergency robotic operations by remote on patients in geographical locations too distant for transfer; or for rare operations where expert surgeons could perform a higher volume of a niche procedure, doing more cases remotely on a global patient pool. For early proof-of-concept studies such as this one to be translated from the bench to the bedside, future independently developed approaches to robotic cardiac surgery are likely to be important for widespread translation into surgical practice. As bioengineers make progress with patches for myocardial regeneration (Menasché et al., 2018; Wang et al., 2021), it is critical that the method of delivery of patches is considered (Roche et al., 2021b). Otherwise is it possible that bioengineers will unveil a new treatment which is limited by the practical consideration of how to transplant it (Roche et al., 2021b). It is important to re-emphasize that the work herein is early stage, because over-inflation of expectations has been identified as a harmful ethical pitfall in the field of regenerative medicine (Gilbert et al., 2018a; Gilbert et al., 2018b; Cossu et al., 2018). Nonetheless, the early-stage proof-of-concept work herein represents a first step on a pathway to achieving minimally invasive robotic cardiac patch transplantation.

## Conclusion and Relevance

For the first time, we have presented a new overall approach to myocardial regeneration—epicardial patch transplantation by minimally invasive robotic surgery. We demonstrated the feasibility of this approach in a surgical operation using a pig cadaver. Our approaches and components are presented freely for others to build upon, without restriction or cost: this has the potential to pivot the field away from its focus on open heart surgery towards a minimally invasive robotic approach—potentially a key step towards clinical translation of myocardial patch transplantation.

## Data Availability

The datasets presented in this study can be found in online repositories. The names of the repository/repositories and accession number(s) can be found below: https://doi.org/10.5281/zenodo.4784952 (CERN, Geneva, Switzerland) with additional datasets in the Supplementary Material attached to this article. All Supplementary Materials were uploaded for peer review with the article.

## Supplementary Material

Supplementary Video S1 Brief overview of robotic keyhole patch transplantation demonstrated with the “Claw” instrument design.

Supplementary Video S2 Video walkthrough for *in silico* demonstration of instrument Control System 1 using potentiometers, an Arduino microcontroller and servo motors.

Supplementary Video S3 Video walkthrough for in silico demonstration of one subunit of Control System 2. The subunit shown is one of three and is tailored to a nine-rod instrument similar to the one we described in our previous preliminary brief research report (Roche et al., 2021b). In the complete control unit the three subunits would be arranged in a wheel-and-spoke formation with 120 degrees between each subunit in the neutral position. The initial ‘sizing and learning’ prototyping result for the full triplicate control unit is shown in Figure 7.

Supplementary Video S4 Detailed background and video walkthrough for the first-in-kind surgical operation to transplant a patch to the heart using the ‘HeartStamp’ instrument. Surgery was performed on a fresh pig cadaver (an appropriate pre-clinical model for cardiothoracic proof-of-concept surgery) and was run once and reported exactly “as-it-happened” (to better simulate “real” surgical conditions). The HeartStamp is referred to as HeartStamp-HPBT (Heart Patch Bearer-Transplanter).

Supplementary Figure S1 The Claw head technical details. The Claw head design would create a secure enclosed space for a patch without crushing it.

Supplementary Figure S2 Outer body design for the “Claw” minimally invasive heart patch transplantation instrument in early-stage development. **(A)** Initial design with rounded-base triangle forming the claw compartment where patch would sit. **(B)** Initial design with tapering rectangular claw compartment. Both initial designs shown here do not completely enclose the patch compartment (a feature of the subsequent final design).

Supplementary Figure S3 Rack and Pinion mechanism technical details.

Supplementary Figure S4 Plate holder technical details.

Supplementary Figure S5 Plate holder working mechanism with combination of linkages. **(A)** L-link **(B)** H-link **(C)** T-link.

Supplementary Figure S6 Shell-Beak Design. First-generation **(A,B)** Shell-Beak design with a patch dispenser system. The cardiac patch is held in an infolded “shell” position and then outfolded with opening of the “beak.” **(C)** External structure **(D)** Internal structure. This instrument was designed to deploy a patch with a diameter larger than the instrument. Elliptical patches with shape-memory could potentially be out-folded and deployed with this design.

Supplementary Figure S7 “Umbrella” Design. Initial designs for a protrusion-based patch deployment instrument which were subsequently developed into the final HeartStamp instrument.
