# Supplementary material for: Cardiac Patch Transplantation Instruments for Robotic Minimally Invasive Cardiac Surgery: Initial Proof-of-concept Designs and Surgery in a Porcine Cadaver
Source: Front Robot AI. 2022 Jan 18;8:714356. doi: 10.3389/frobt.2021.714356 (PMC8804503; doi:10.3389/frobt.2021.714356)
Supplement: Supplementary file 1 [file DataSheet2.pdf]

Non-Author Contributors

**Anuj Kumar**	Master of Professional Engineering (Mechanical)
**Tina Pun**	Master of Professional Engineering (Biomedical)
**Xinhang Liu**	BEng (Mechanical Engineering) (Hons)
**Nan Zhao**	Master of Professional Engineering (Mechanical)
**Abinas Rai**	BEng (Mechanical Engineering) (Hons)
**Harris Harly**	BEng (Mechanical Engineering) (Hons)
**Anmol Muralidhar**	Master of Professional Engineering (Biomedical)
**Parth Kanani**	Master of Professional Engineering (Mechanical)
**Alfa Fernandes**	BEng (Mechanical Engineering) (Hons)
**Sarbartha Goswami**	Master of Professional Engineering (Mechanical)
**Liang Zhao**	Senior Lecturer, School of Mechanical and Mechatronic Engineering, Faculty of Engineering and IT, UTS. Supervisor of Yiran Zhou.
**Yiran Zhou**	Recently graduated final year UTS mechanical engineering student BEng (Mech) (Hons) supervised by Liang Zhao.
**Robert Lewi**	UTS Casual Academic (Optik Summer Internship)

Contributions

Anuj Kumar – Investigated the feasibility of any ideas thought of by the group and suggesting alternatives if ideas are proven to be impossible, from a mechanical aspect. He also looked into the possible mechanism to operate the device such as Endo wrist and DaVinci system. He, alongside Gautam Ranganathan Iyer, investigated the master-slave control system that can be applied to Arduino microcontroller for controlling the surgical instrument in a semi-autonomous method. He also assisted in decrypting the invert-umbrella design to understand its working mechanism, so as to provide the team with sufficient information to develop a control system. Conducted literature review by researching articles on connecting the control system to the instrument design.

Tina Pun – Investigated minimal invasive instruments and the technology already existing in the medical field. She and Sohaima Mabroora researched on cardiac patch delivery system by comparing the minimally invasive cardiac surgery with laparoscopic surgery. She brainstormed design ideas with Abinas Rai to develop the Shell Beak Design for the patch delivery instrument. She reviewed the control mechanism being used in robotic laparoscopic surgical instruments. She also conducted a risk management study for minimally invasive instruments, that assisted the designing team to mitigate possible design risks during development of Claw design, Shell Beak design, and Umbrella design. Looked into the sterilization requirement for the surgical instrument with Gautam Ranganathan Iyer and proposed the idea of having a disposable instrument.

Xinhang Liu – Developed the Umbrella Design in SolidWorks based on the inverted umbrella design concept. Created a prototype with the help of Sohaima Mabroora using straws, paper, and rubber bands to replicate the motion so that she can study and showcase the mechanics. She conducted a literature review for the working mechanism of the umbrella design. Based on the research investigated, Minh Huy Nguyen helped her to re-design the SolidWorks file. She, alongside Sohaima Mabroora, investigated and developed the coding and simulation of the control system using Arduino microcontroller. She also compared the financial forecasting for prototyping the claw design.

Nan Zhao – Developed the internal part (shell) for the Shell-Beak Design in SolidWorks with Abinas Rai. Analysed the invert umbrella design and derived the mechanism to develop the control system. He used SolidWorks to 3D model the motors involved in the control system to be used for Claw Design. He created a motion study by mounting (connecting the control system onto the rear end of Claw

Design) the servo motors (device that facilitates the linear motion for rods in the claw design. This is a part of the control system) on to the claw design and simulating them.

Abinas Rai – Developed the external part (beak) for the Shell-Beak design in SolidWorks with Nan Zhao. He created a motion study to simulate the working of this design. Along with Gautam Ranganathan Iyer, he researched methods to optimize the control system model and assisted to write the Arduino coding for the claw design using Tinker CAD software. He also researched on the types of motors that can be used to power the control system and 3D model it in SolidWorks which is added to the design.

Harris Harly – Led the team on developing a control system of client's instrument. Contributed on turning the sketches, ideas, comments, and feedbacks from Parth Kanani and Sarbartha Goswami into 3D Drawing by using Solid works. He had experience on 3D printing and did research on suitable materials for the designed control system. He identified each of the instrument's components by using FEA test to make sure the instrument can hold the load of 10 N.

Anmol Muralidhar – Development of design of the instrument, about the biomaterials and standards used for medical equipment's. Also, researched on inverse kinematics and roto linear actuators for the movement of rods inside the instrument. Researched on cardiac patch and its composition to analyze suitable design mechanism for holding it. Designed a Gantt chart to identify and track contributions of each of us during our internship.

Parth Kanani – Sketched first idea of control system and specified the total degree of freedom. Also, designed the control mechanism for cardiac patch surgical instrument which operate end effector of the instrument. He identified total motions of each connecting rods which was connected by instrument with help of team members Harris Harly and Alfa Fernandes. He designed the first idea of control mechanism which includes 2 motors and 3 linear actuators. In support of other team members Sarbartha Goswami and Anmol Muralidhar, he analyzed the minimum least motion of instrument which should not affect the internal organs of the body. According to restriction which they have considered, he defined some constraints to set limits of motion under safety of human body.

Alfa Fernandes – Designed the control mechanism given by Parth Kanani in SolidWorks part. Investigated the working of invert umbrella design of the instrument. Brainstormed ideas for the control system of the instrument. Assisted other team member designing their design in SolidWorks and fixed some errors. Designed a control system for design 3.

Sarbartha Goswami – Designed the mechanism of achieving multi directional rotation along the same axle and analyzed the compatibility to implement maximum number of actuators in a fully automated robotic instrument designed for delivering cardiac patch. Investigated the required motion (linear and rotational) to operate the instrument precisely during a heart surgery. Designed working mechanism to mimic 12 degree of freedom (DOF) motion with the help of gear train, linear actuators, and servo motors. Analyzed the mechanical and biomedical constraints of the project with Parth Kanani and Anmol Muralidhar. Assisted in developing a 3D model for the rotational and linear motion mechanism built by Harris Harly and Alfa Fernandes in SolidWorks. Researched about the displacement, accelerometer, and force sensors needed for the rotational and linear motion mechanism and respective control theory.

Robert Lewi – Advised the students ("Optik" summer student internship students as listed below) working on the instrument and control system in matters of presentation of their research and project management.

Yiran Zhou and Liang Zhao – Advised students and provided administrative and supervisory input at meetings (based on their previous work on the project with Christopher D Roche and Carmine Gentile – data not used in this manuscript – prior to the work of the "Optik" Summer Interns).
