# Supplementary figures and images for: Cardiac Patch Transplantation Instruments for Robotic Minimally Invasive Cardiac Surgery: Initial Proof-of-concept Designs and Surgery in a Porcine Cadaver
Source: Front Robot AI. 2022 Jan 18;8:714356. doi: 10.3389/frobt.2021.714356 (PMC8804503; doi:10.3389/frobt.2021.714356)

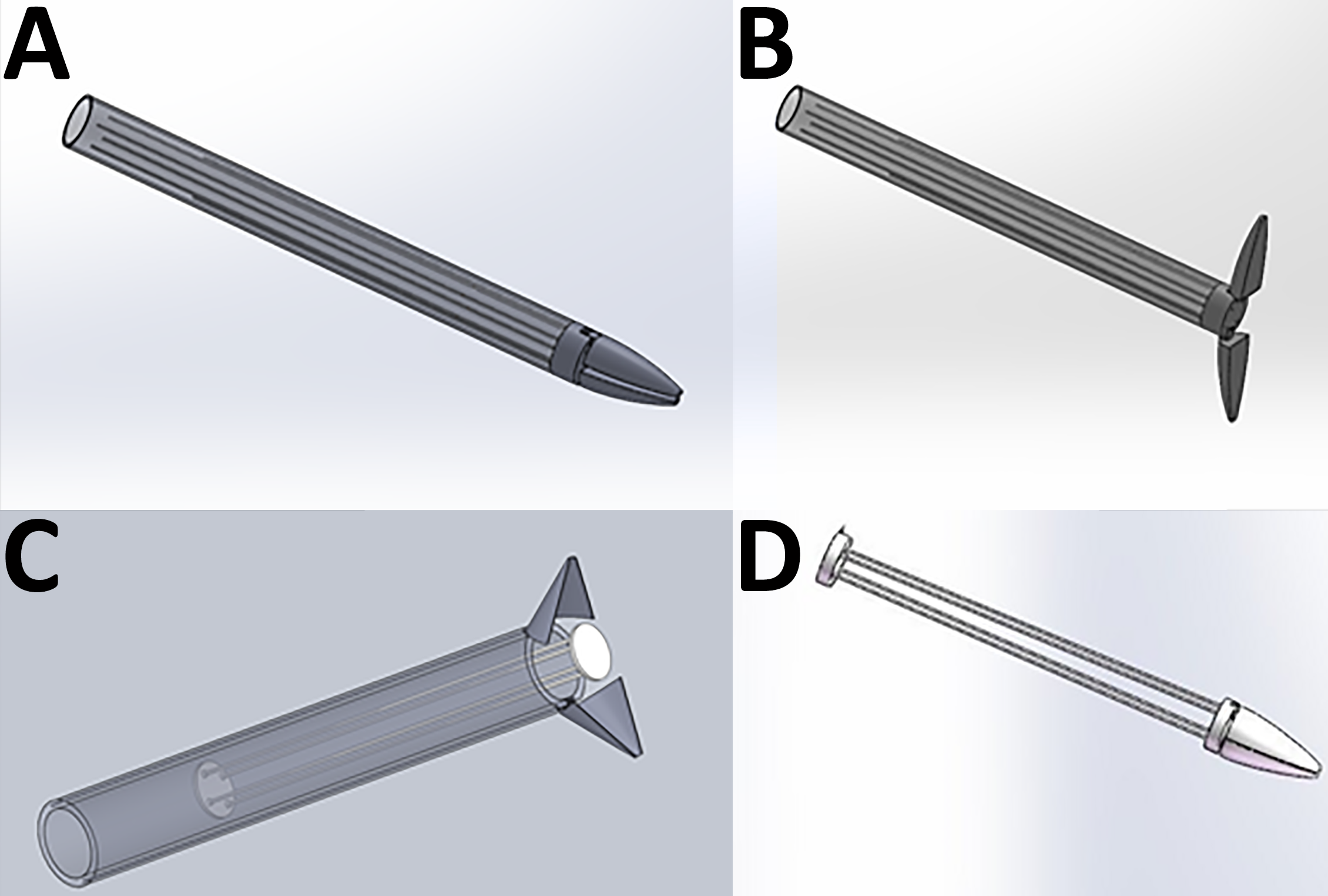

Supplement: Supplementary file 2 [file Image6.TIF]

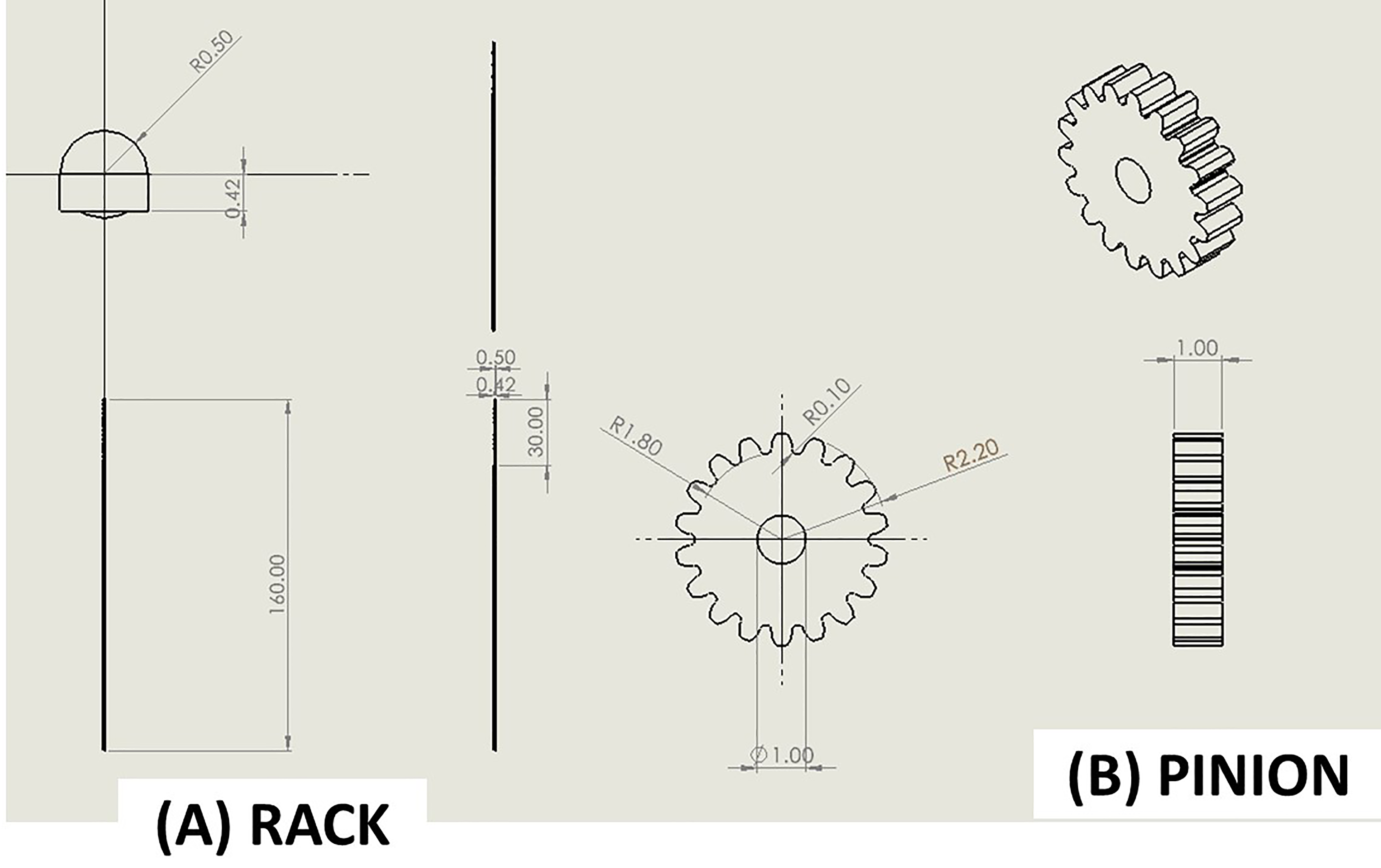

Supplement: Supplementary file 4 [file Image3.TIF]

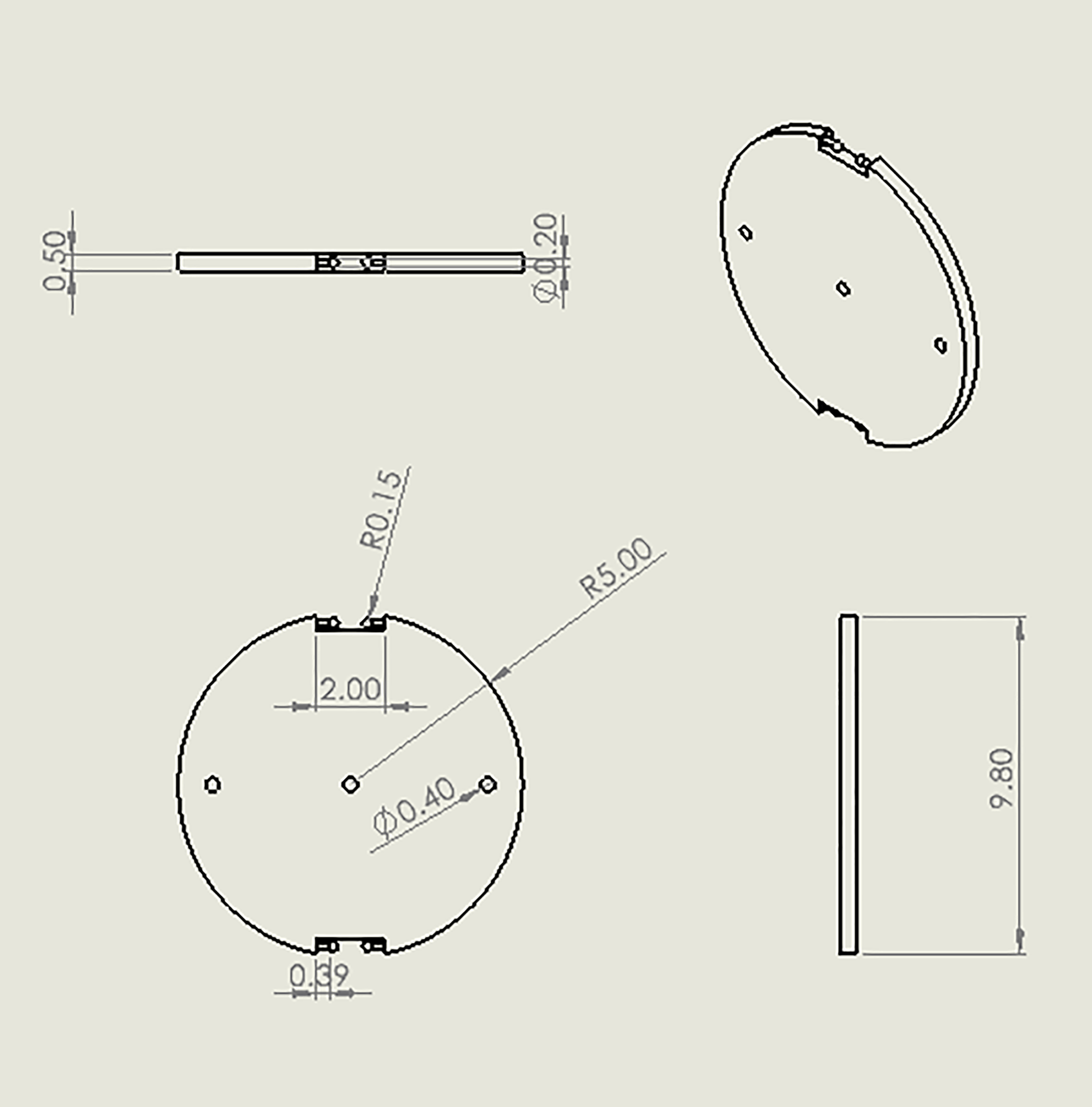

Supplement: Supplementary file 5 [file Image4.TIF]

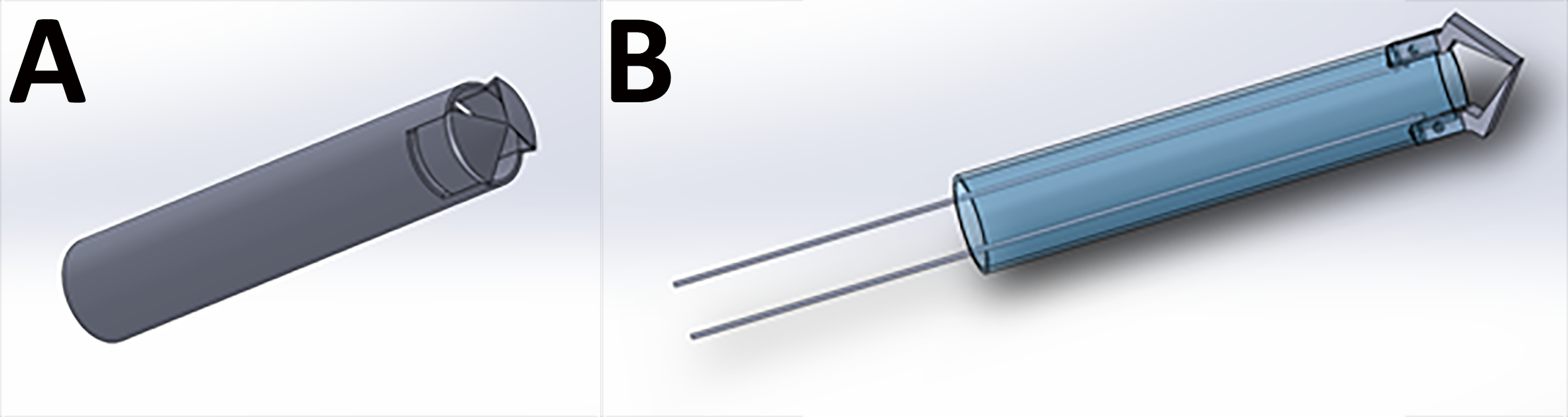

Supplement: Supplementary file 8 [file Image2.TIF]

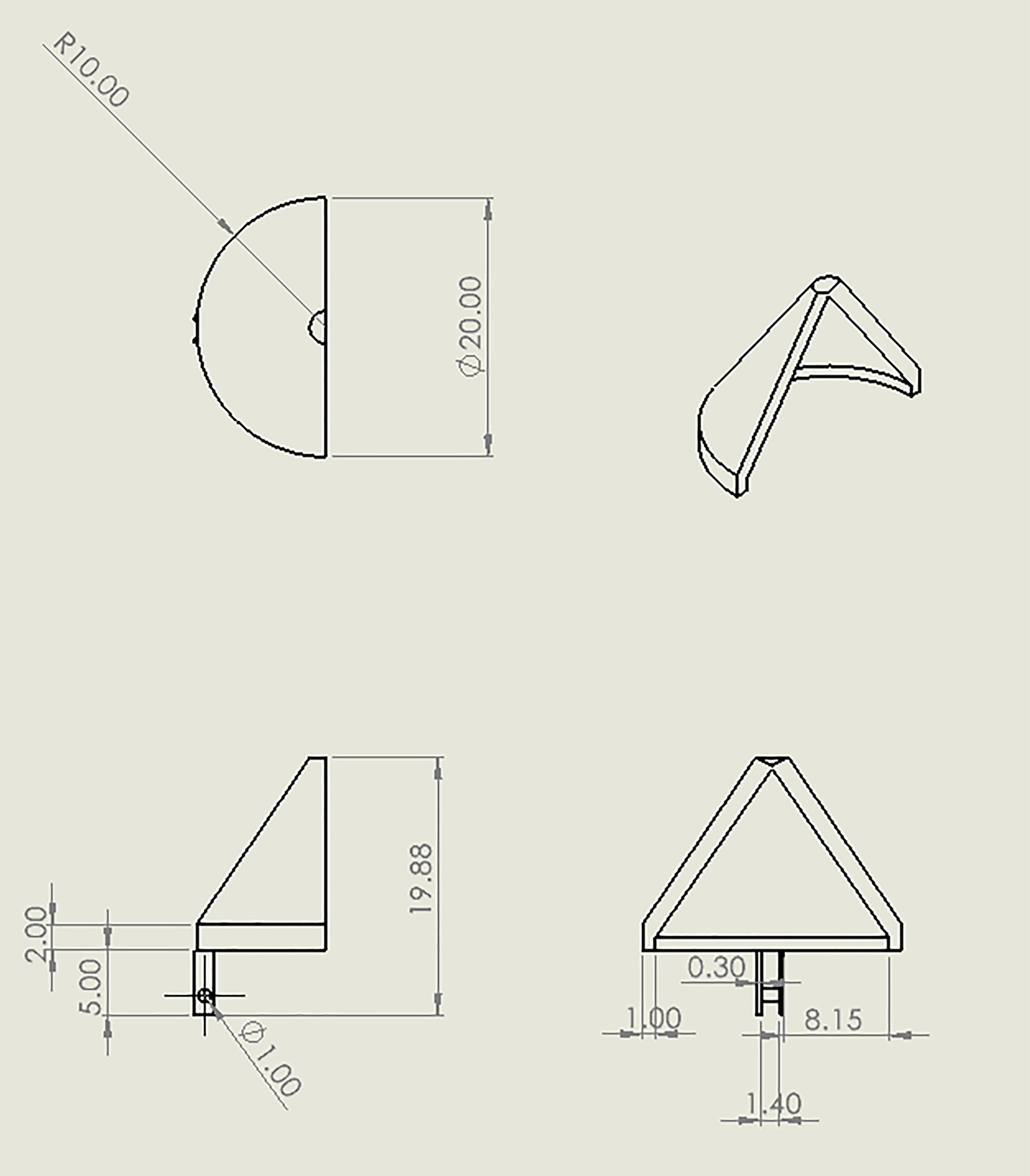

Supplement: Supplementary file 9 [file Image1.TIF]

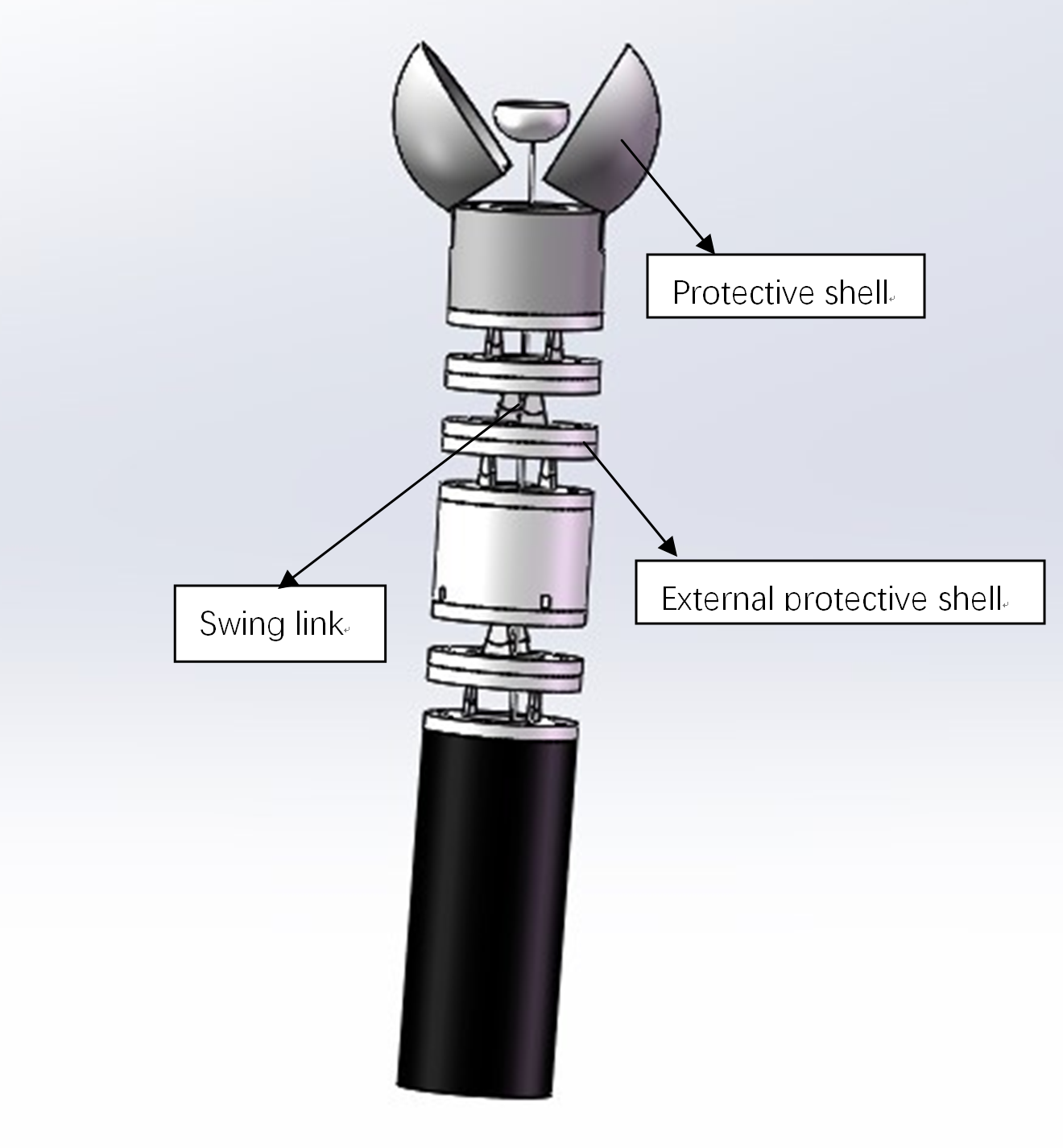

Supplement: Supplementary file 10 [file Image7.PNG]

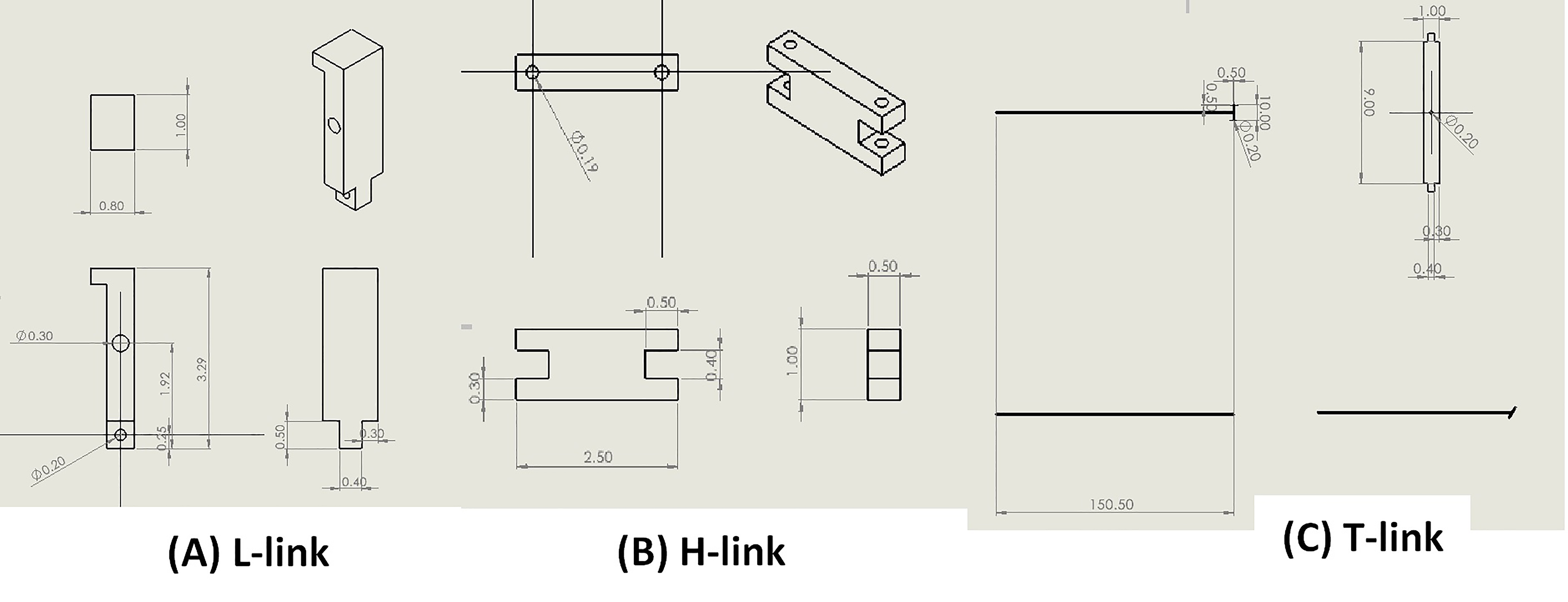

Supplement: Supplementary file 13 [file Image5.TIF]
