# Supplementary material for: Cardiac Patch Transplantation Instruments for Robotic Minimally Invasive Cardiac Surgery: Initial Proof-of-concept Designs and Surgery in a Porcine Cadaver
Source: Front Robot AI. 2022 Jan 18;8:714356. doi: 10.3389/frobt.2021.714356 (PMC8804503; doi:10.3389/frobt.2021.714356)
Supplement: Supplementary file 6 [file DataSheet3.docx]

بافت های اسیب دیده قلب می تواند به طور بالقوه با پیوند تکه های قلبی مهندسی شده زیستی به سطح قلب بازسازی شوند و بافت های اسیب دیده ی قلب را ترمیم کنند. برای تغییر الگوی کامل، چنین تیکه هایی ممکن است نیاز به پیوند با استفاده از جراحی قلب رباتیک کم تهاجم که ریسک کمتری در مقایسه با عمل جراحی قلب باز دارد استفاده شود. طرح یا روش این ازمایش اینگونه بود که اول ابزارهای عمل جراحی راباتیک و سیستم های کنترل خودکار طراحی شدند و نمونه اولیه شدند.برای اثبات این ازمایش بر روی جسد خوک انجام شد و سه طرح ابزار رباتیک توسعه داده شد.

اولین طرح *claw* است که بر روی قفسه و میکانیسم *pinion* عمل می کند. طرح دوم *shell beak* نامیده میشود که از صفحات تاشو قابل تنظیم و میله های چرخ دنده استفاده می کند. طرح سوم *heart stamp* است از *stamp platform* که از طریق یک حلقه قابل تنظیم بیرون زده استفاده می کند. در طرح سوم، میله ها از یک ساختار استواره ای عبور می کنند که متناسب با یک پورت جراحی توراسکوپی با کمک یک ویدئو (VATS) طراحی شده است. این وصله که برای کار با و یا بدون *sterile sheath* طراحی شده است، هنگام بیرون زدگی توسط *stamp platform* به بیرون رانده می شود. دو سیستم کنترل رباتیک ابزار طراحی شدند که یکی از این ها به عنوان اثبات مفهوم اولیه تحت اندازه گیری و یادگیری قرار گرفت. برای انعکاس شرایط واقعی جراحی، عمل به صورت زنده اجرا شد و سپس به طور دقیق گزارش شد. ما با موفقیت *patch* را با استفاده از *heart stamp* توانستیم به روی قلب انتقال دهیم. روش جراحی رباتیک قلب نه تنها از نظر هزینه مناسب تراست بلکه افرادی که برای انجام عمل قلب باز مناسب نیستند می توانند از این روش که از ریسک کمتری برخوردار است سلامتی قلب خود را به دست بیاورند.
