# Supplementary material for: Cardiac Patch Transplantation Instruments for Robotic Minimally Invasive Cardiac Surgery: Initial Proof-of-concept Designs and Surgery in a Porcine Cadaver
Source: Front Robot AI. 2022 Jan 18;8:714356. doi: 10.3389/frobt.2021.714356 (PMC8804503; doi:10.3389/frobt.2021.714356)
Supplement: Supplementary file 15 [file DataSheet1.docx]

**Supplementary Materials: Methodological (Process) Details**

**I) Scamper table to brainstorm ideas to develop the HeartStamp device**

| Substitute​ | Combine​ | Adapt​ | Modify/Magnify​ | Put to another use​ | Eliminate​ | Reverse/Rearrange​ |
| --- | --- | --- | --- | --- | --- | --- |
| Substitute the materials for all designs from 3dprinted plastic to a range of more basic materials found in hardware stores​ | We combined the ring design with the flat plate design​ | We adapted the flat plate from being a device to hold the patch to a device that only forces the patch onto the heart​ | We increased the size of all the ideas to increase the thickness of the parts (especially with the thin rods)​ | N/A​ | From the umbrella design we can eliminate many joints and the design can still function ​ | We rearranged the flat plate to also be part of the control interface​ |

**II) Tree diagram to develop the concept of HeartStamp device**


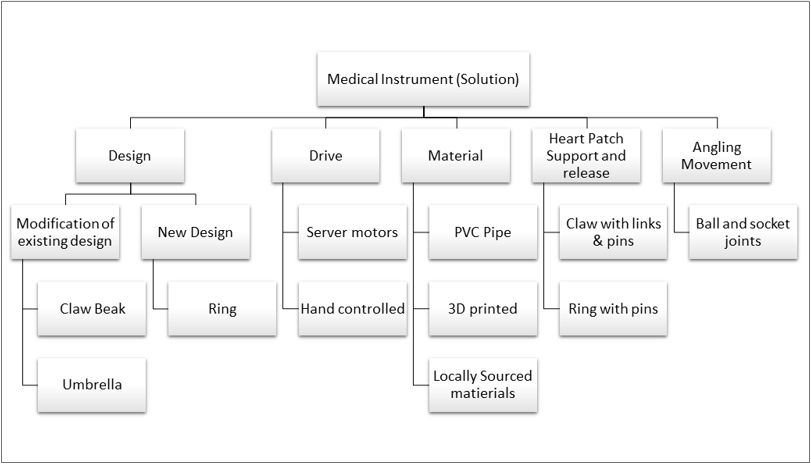


**III) Materials used for individual parts for developing the HeartStamp device prototype**

| **Part** | **Material** |
| --- | --- |
| Centre Rod | Stainless steel |
| Puck | Rubber |
| Metal Ring | Stainless steel |
| Support | PEEK (desired), For prototype timber was used for cost measures |
| Tube | Plastic |
| Ring Rod | Stainless Steel |
